# Structural and functional connectivity associations with anterior cingulate sulcal variability

**DOI:** 10.21203/rs.3.rs-3831519/v1

**Published:** 2024-01-05

**Authors:** Luke Harper, Olof Strandberg, Nicola Spotorno, Markus Nilsson, Olof Lindberg, Oskar Hansson, Alexander F Santillo

**Affiliations:** Lund University; Lund University; Lund University; Lund University; Karolinska Institute; Lund University; Lund University

**Keywords:** Cingulate, Paracingulate, Sulcation, Resting-state, Functional Connectivity

## Abstract

**Background:**

Sulcation of the anterior cingulate may be defined by presence of a paracingulate sulcus, a tertiary sulcus developing during the third gestational trimester with implications on cognitive function and disease.

**Methods:**

In this retrospective analysis we examine task-free resting state functional connectivity and diffusion-weighted tract segmentation data from a cohort of healthy adults (< 60-year-old, n = 129), exploring the impact of ipsilateral paracingulate sulcal presence on structural and functional connectivity.

**Results:**

Presence of a left paracingulate sulcus was associated with reduced fractional anisotropy in the left cingulum (*P* = 0.02) bundle and the peri-genual (*P* = 0.002) and dorsal (*P* = 0.03) but not the temporal cingulum bundle segments. Left paracingulate sulcal presence was associated with increased left peri-genual radial diffusivity (*P* = 0.003) and tract volume (*P* = 0.012). A significant, predominantly intraregional frontal component of altered resting state functional connectivity was identified in individuals possessing a left PCS (*P* = 0.01). Seed-based functional connectivity in pre-defined networks was not associated with paracingulate sulcal presence.

**Conclusion:**

These results identify a novel association between neurodevelopmentally derived sulcation and altered structural connectivity in a healthy adult population with implications for conditions where this variation is of interest. Furthermore, they provide evidence of a link between the structural and functional connectivity of the brain in the presence of a paracingulate sulcus which may be mediated by a highly connected local functional network reliant on short association fibres.

## Introduction

**Figure 1:**
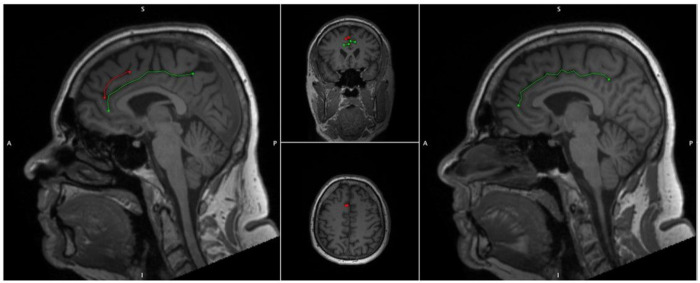
Cingulate and Paracingulate Sulci Identification and Measurement 52-year-old male displaying a Leftward pattern of paracingulate asymmetry. Left panel, a sagittal slice of the left hemisphere with a traced “present” (length ≥20mm), left paracingulate sulcus (red) and a traced left cingulate sulcus (green). Right panel, a sagittal slice of the right hemisphere, the paracingulate sulcus is absent here and only the cingulate sulcus is traced (green)

The Anterior Cingulate (AC) is a highly heterogenic medial frontal lobe gyrus with extensive interindividual variability and asymmetry. Variability may be classified in accordance with the presence of a Paracingulate Sulcus (PCS), a tertiary sulcus, which when present develops during the third trimester of gestation and remains stable thereafter, unaffected by maturation or environmentally induced neuroplastic changes (Chi, Dooling et al. 1977, Del Maschio, Sulpizio et al. 2019). The PCS denotes the existence a respective Paracingulate Gyrus (PCG). In healthy individuals there is an established leftwards-dominance of PCS presence (presence of a left, but not right hemisphere PCS), as displayed in Fig. 1. (Paus, Tomaiuolo et al. 1996, Yücel, Stuart et al. 2001, Yücel, Stuart et al. 2002, Le Provost, Bartres-Faz et al. 2003, Huster, Westerhausen et al. 2007, Leonard, Towler et al. 2009, Wei, Yin et al. 2017, Amiez, Wilson et al. 2018, Selahi, Kuru Bektaşoğlu et al. 2022). Whilst reported frequencies vary PCS are present in approximately 70–75% of left hemispheres and 50–60% of right hemispheres in the healthy population (Paus, Tomaiuolo et al. 1996, Yücel, Stuart et al. 2001, Fornito, Yücel et al. 2004). The PCG is active during performance of a variety of cognitively demanding tasks drawing on higher-order executive function. (Fornito, Yücel et al. 2004) (Carter et al., 1998; Duncan and Owen, 2000). A performance advantage across several verbal and non-verbal higher-order functions utilising effortful cognitive control and verbal and spatial working memory has been observed in individuals possessing a leftward asymmetry of PCS presence (Fornito, Yücel et al. 2004) (Whittle, Allen et al. 2009). Similarly, individuals with asymmetric PCS patterns display greater inhibitory control and cognitive efficiency than those with symmetric patterns (Tissier, Linzarini et al. 2018) (Borst, Cachia et al. 2014) (Huster, Wolters et al. 2009) (Cachia, Borst et al. 2014) (Fedeli, Del Maschio et al. 2022). Bilateral PCS absence is considered cognitively disadvantageous and is associated with reduced reality monitoring and performance related introspection (Buda, Fornito et al. 2011).

In disease PCS variability has been associated with schizophrenia and behavioural variant Frontotemporal Dementia (bvFTD), both of which have known pathological foci in the anterior cingulate. In bvFTD presence of a right PCS is associated with disease expression and survival (Harper, Lindberg et al. 2022, Harper, de Boer et al. 2023), whilst a reduced frequency of leftward paracingulate sulcation dominance is observed in individuals with schizophrenia (Yücel, Stuart et al. 2002, Le Provost, Bartres-Faz et al. 2003, Yücel, Wood et al. 2003, Marquardt, Levitt et al. 2005, Fujiwara, Hirao et al. 2007). Furthermore, left hemisphere PCS presence is reportedly less frequently in patients with obsessive compulsive disorder (OCD)(Shim, Jung et al. 2009) and altered PCG connectivity has been correlated with generalized epilepsy (Kay, Holland et al. 2014) and epilepsy drug resistance (Szaflarski, Kay et al. 2013) (Wysiadecki, Mazurek et al. 2021).

There are several ways in which PCS presence may mediate function and thus its role in disease. Cortical folding, according to the tension-based morphogenesis theory is considered to be pathway specific, partially dependent on underlying tensions between short association fibres connecting neighbouring cortical regions which shorten to reduce wiring (Van Essen 1997, Van Essen 2020). Reflecting this process, the PCG primarily contributes U-fibres connecting it with the AC proper which forms a localised white matter network not present where a PCS is absent (Wysiadecki, Mazurek et al. 2021). There fibres may influence the observed underlying cytoarchitectural differences of the AC observed in the presence of a PCS (Vogt, Nimchinsky et al. 1995).

Deep of the superficial U-fibres lie longitudinal fibres which course within the PCG when present and are identified medial and slightly inferior to the cingulate sulcus, within the cingulate gyrus where the PCS is absent (Komaitis, Skandalakis et al. 2019). This tract is regarded by some as subcomponent Ia of the superior longitudinal fasciculus (SLF), (Komaitis, Skandalakis et al. 2019) (Wysiadecki, Mazurek et al. 2021) and by others as a division of the cingulate bundle (Wu, Sun et al. 2016) or even U-fibres (Maldonado, Mandonnet et al. 2012). To the best of our knowledge no neuroanatomical tracing or tractography studies have been performed with respect to PSC presence. From the perspective of intrinsically connected networks, the SLF-I is considered a major subcortical connection of the default mode network (DMN), a resting state network activated when the brain is resting (but alert) and attention is focused on internal tasks such as memory retrieval and self-reflection (Yagmurlu, Middlebrooks et al. 2016). Moreover, the AC contains the cingulum bundle and represents a key hub of the Salience network (SN) (Seeley, Menon et al. 2007). Operationally, the SN processes relevant stimuli by integrating sensory, emotional, and cognitive information, becoming active during tasks requiring attentional selection, task switching, and self-regulation of behavior (Farb, Grady et al. 2013) (Fedeli, Del Maschio et al. 2020) (Seeley, Menon et al. 2007).

Presence of a PCS has been identified to alter the loci of task-based functional connectivity in numerous works (Jahn, Nee et al. 2016) (Amiez, Neveu et al. 2013). To date only one study (Fedeli et al 2020) has examined resting-state functional connectivity with respect to the PCS, identifying an association with functional connectivity in target voxels overlapping components of the SN and DMN but without a convincing pattern emerging (Fedeli, Del Maschio et al. 2020). The impact of PCS presence on resting state functional connectivity remains incompletely explained and has not, to the best of our knowledge been studied alongside structural connectivity in the same cohort. In the present study we examine task-free resting state functional data and diffusion-weighted tract segmentation data in a cohort of young adults (< 60-year-old), exploring the impact of PCS presence on structural and functional connectivity.

With respect to the tension-based morphogenesis hypothesis of gyrification, (Van Essen 1997, Van Essen 2020) structurally we hypothesise that presence of a PCS shall alter the SLF-I and/or cingulum bundle tracts both at a macroscopic (i.e., volume of the tracts) and microscopic (i.e., using diffusion tensor imaging metrices as proxies) level reflecting different local structural connectivity in individuals possessing a PCS. Functionally, we hypothesise that presence of a PCS shall be associated with a highly localised network component and that increased network connectivity strength shall be observed in predefined local networks (the SN and DMN).

## Materials and Methods

### Participants

In this retrospective analysis we studied data from healthy subjects from the Swedish BioFINDER-2 study (Skåne University Hospitals, Sweden [NCT03174938]), which was approved by the Regional Ethical Committee in Lund, Sweden, (EPN file number 2016/1053). Participants were enrolled between 2014 and 2021 following attainment of written consent in accordance with the Declaration of Helsinki. For further study details, see http://biofinder.se and (Palmqvist, Janelidze et al. 2020). Briefly, study participants were recruited using the following inclusion criteria: (i.) absence of cognitive symptoms, (ii.) Mini-Mental State Examination (MMSE) score of 26–30 at baseline, (iii.) not fulfilling criteria for mild cognitive impairment or dementia according to DSM-5 (American Psychiatric Association 2013), (iv.) absence of active psychological or psychiatric disease and (v.) fluency in Swedish. Additional exclusion criteria used in the present study were: (i.) age ≥ 60 years old, (ii.) an abnormal CSF amyloid-ß42/40 ratio, described in the **Supplementary Material**, (iii.) a high volume of white matter hyperintensities, (> 3 standard deviations from the cohort mean) identified as described in the **Supplementary Material**, and (iv.) poor MRI image quality obscuring identification of the PCS.

### Magnetic Resonance Image Acquisition

MRI scans were performed on a MAGNETOM Prisma 3T scanner (Siemens Healthineers, Erlangen, Germany), equipped with a 64-channel head coil. A T1w MPRAGE (magnetization-prepared rapid gradient-echo) sequence was acquired with the following acquisition parameters: repetition time: 1900 ms; echo time: 2.54 ms; echo spacing: 7.3 ms; voxel size: 1×1×1 mm^3^ and field of view: 256×256×176 mm^3^. GRAPPA (generalized autocalibrating partially parallel acquisitions^33^) was applied with acceleration factor of 2 and 24 reference lines. A single-shot echo-planar imaging sequence was used to acquire 104 diffusion-weighted imaging volumes (repetition time: 3500 msec; echo time: 73 msec; resolution: 2×2×2 mm^3^; field of view 220×220×124 mm^3^; b values range: 0, 100, 1000, and 2500 sec/mm^2^ distributed over 2, 6, 32, and 64 directions; twofold parallel acceleration and partial Fourier factor=7/8). A second diffusion MRI scan was also obtained with a reverse phase-encoding and 7 gradient directions (1 × b = 0 and 6 × b = 1000 s/mm^2^) for correction of susceptibility-induced distortions. A T2-weighted FLAIR scan (repetition time: 5000; echo time 393 ms, same resolution and FoV as for the T1-weighted image) was also acquired. Spontaneous blood oxygen level-dependent (BOLD) oscillations were acquired with a gradient-echo planar sequence (eyes closed, in-plane resolution = 3 × 3 mm2, slice thickness = 3.6 mm, repetition time = 1020 ms, echo time = 30 ms, flip-angle = 63°, 462 dynamic scans, 7.85 min)

### Paracingulate Sulcus Measurement and Classification Criteria

Individuals were grouped in accordance with hemispheric presence of a PCS. PCS presence was identified via manual classification of structural T1 MRI data according to an adapted version or Garrison’s established protocol for PCS classification (Garrison, Fernyhough et al. 2015), which has been used and described previously (Harper, Lindberg et al. 2022, Harper, de Boer et al. 2023) and is documented in full in the **Supplementary Material.** Briefly, potential PCS, meeting classification criteria were manually traced and measured. As is standard amongst classification protocols hemispheres with a PCS ≥ 20mm in length were categorised as possessing a “present” PCS whereas hemispheres failing to meet these criteria were deemed to possess an “absent” PCS (Ono, Kubik et al. 1990, Yücel, Stuart et al. 2002, Le Provost, Bartres-Faz et al. 2003, Garrison, Fernyhough et al. 2015, Del Maschio, Sulpizio et al. 2019). Sulcation ratings were performed independently by two raters, LH and AS, who were blinded to individuals’ demographic data. Disagreement between raters was resolved by consensus.

### MRI data processing

MPRAGE images were imported into MANGO (Multi-image Analysis GUI, v 4.0, http://ric.uthscsa.edu/mango/mango.html, The University of Texas Health Science Center) software and prepared, aligning the x axis in the sagittal plane with the bicommissural line (AC–PC). Further y and z axis rotational corrections were performed in order to ensure optimal orientation for analysis.

### Tract Segmentation

**Figure 2:**
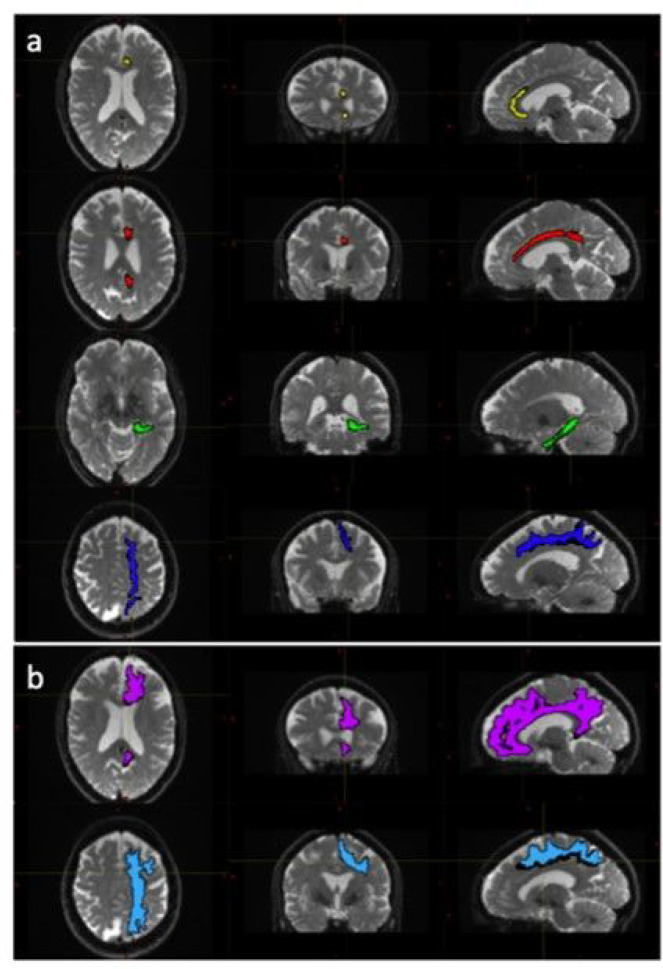
Tract Segmentations of the cingulate bundle and superior longitudinal fasciculus Diffusion weighted image data for a 53-year-old female participant with left hemisphere tract segmentation mask overlays. Panel (a) displays the cingulum, divided into the peri-genual (yellow), dorsal (red) and temporal (green) tracts and the superior longitudinal fasciculus (dark blue), extracted according to the Xtract definition. Panel (b) displays the cingulum bundle (pink) and the superior longitudinal fascicules (light blue) extracted according to the Tract Seg definition

The diffusion weighted data were processed using a combination of open-source algorithms. In brief the acquired images were denoised and the Gibbs ringing artifacts were removed using *MRtrix3* (Tournier, Smith et al. 2019) routines. Correction for susceptibility induced distortions, using images acquire with opposite phase polarities, motion and Eddy currents was performed employing *FSL Top-up* (Andersson, Skare et al. 2003) and *Eddy* (Andersson and Sotiropoulos 2016) (FMRIB Software Library, version 6.0.4; Oxford, United Kingdom). Parametric maps of mean diffusivity (MD), fractional anisotropy (FA), axial diffusivity (AD) and radial diffusivity (RD) were computed using DIPY (Henriques, Correia et al. 2021) routines (https://dipy.org/) Following pre-processing of diffusion MRI scans, white matter tracts were segmented using, TractSeg (Wasserthal, Neher et al. 2018). Both the 72 tracts definition included in TractSeg and the 42 tracts definition derived from Xtract (Warrington, Bryant et al. 2020) were used in order to improve internal validity. Furthermore, the Xtract method divides the cingulate bundle into three distinct tracts offering a more focused analysis of white matter contiguous with the PCS. Diffusivity metrics and tract volumes were analysed in accordance with ipsilateral hemispheric PCS presence in the superior longitudinal fasciculus I (SLF-I) [both segmentation methods], the cingulum (CG) [TractSeg] and the dorsal (CBD), pre-genual (CBG), and temporal (CBT) cingulum [Xtract]. Further method description and quality control measures are documented in the **Supplementary Material**. Tract segmentations example are displayed in Figure 2.

### Resting State Functional MRI pre-processing

Resting state functional MRI data pre-processing was performed using a pipeline composed of FSL(Jenkinson, Beckmann et al. 2012), AFNI(Cox 1996) and ANTS(Avants, Tustison et al. 2014). Anatomical processing involved skull stripping, segmentation of CSF, white and grey matter, and normalization to MNI152 space(Grabner, Janke et al. 2006). Following bulk motion and slice timing correction, nuisance regression compensated white matter/CSF signal, physiological noise(Behzadi, Restom et al. 2007), motion parameters(Johnstone, Ores Walsh et al. 2006), and scanner drift. Finally, the functional data were band-pass filtered (0.01–0.1 Hz) and transformed to MNI space. Frames causing outliers in total frame-to-frame signal variation (75 percentile + 1.5 interquartile range) were censored (Power, Barnes et al. 2012). Subjects with a mean/maximum framewise displacement exceeding 0.3/3.0mm were excluded. The processed functional MRI data were resampled to 6 × 6 × 6 mm^3^ and masked with grey matter derived from a cortical resting-state network atlas (Thomas Yeo, Krienen et al. 2011), Harvard-Oxford subcortical atlas (Desikan, Ségonne et al. 2006). The variance stabilized Fisher z-transformed Pearson correlation between the resulting grey matter BOLD voxel time series yielded our functional connectivity measure.

### Statistical Analysis

#### Tract Segmentation analysis

Diffusivity metrics and tract volume analyses were performed in R software (R Version 4.2.1 CoreTeam 2016, https://www.r-project.org/) using general linear models, including age, sex, and handedness as covariates in all models. In addition, individual’s total intracranial volume was included as a covariate in all models analysing tract volume. As these analyses were explorative correction for multiple comparisons was not performed.

#### Seed-based Functional Connectivity analysis

The Salience/Ventral Attention, Default mode and Visual networks were defined geographically according to network parcel locations defined by the Schaefer 200 parcel 7 network atlas (Schaefer, Kong et al. 2017), further descriptions are provided in the **Supplementary Material**.

Functional connectivity (FC) analysis was performed using Pearson correlation coefficients between the mean time series of the 200 seeds corresponding to the 200 parcels of the Schaefer 200 parcel 7 network atlas. FC’s were converted into z-scores to improve normality using Fisher r-to-z transformation. Individuals z-scores were then averaged across ROIs relating to the predefined networks of interest. Finally, GLMs were fitted according to group averaged z-scores determined by ipsilateral PCS presence, controlling for the effects of age, sex, and handedness. Significance was identified at *P* = 0.05.

#### Voxel-based Functional Connectivity analysis

A medial frontal lobe region of interest (ROI) was created for each hemisphere using the Schaefer 200 parcel 7 network atlas (Schaefer, Kong et al. 2018). Selected parcels were those overlapping the predicted location of the PCS in MNI-152 space (Grabner, Janke et al. 2006). ROIs are detailed in **Supplementary Figure 1**.

Voxel wise whole brain connectivity in 6 × 6 × 6 mm^3^ space was evaluated using a two-step procedure. First the mean connectivity of all voxels was calculated using Persons r correlation.

**Figure 3:**
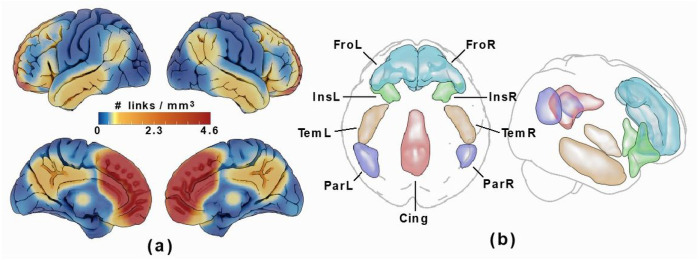
Voxel-based Connectivity Results Cortical ROI set with high connectivity to the medial frontal lobe ROI, (visualised in **Supplementary Figure 2**). Panel (a) shows the link density from the medial frontal lobe ROI used to manually delineate highly connected regions in panel (b): frontal (Fro: overlaps cing ant/mid, front sup med, front sup/mid, front mid/med/inf/sup orb), insular (Ins: overlaps insula, temp pole mid/sup, front inf orb/tri), temporal (Tem: overlaps temp inf/mid, temp pole mid), parietal (Par: overlaps par inf, temp mid/sup, angular, supramarginal) and posterior cingulate (Cing: overlaps cing mid/post, precuneus). To these, two subcortical ROIs defined in the Harvard-Oxford subcortical atlas were added (not shown): basal ganglia (BG: thalamus, Caudate, Pallidum, Putamen) and hippocampus/amygdala (HiAm)

The functional connectome was then restricted with a network mask corresponding to high connectivity with the medial frontal lobe ROIs by thresholding the all-subject-mean connectivity of all subjects at a correlation corresponding to *P* = 0.0001 (given the number of frames in the rs-fMRI time series). Cortical ROIs corresponding to this network mask, which included the bilateral anteromedial frontal cortices as well as portions of the insular, lateral temporal, parietal, and posterior cingulate cortices were then drawn on the resulting voxel-wise link density maps, see Figure 3. These regions are part of the DMN and SN resting state networks, which both overlap with the source region. As scattered connectivity was obtained with subcortical regions of the basal ganglia and hippocampus/amygdala, these structures were added to the ROI set using the anatomical definitions according to the Harvard-Oxford subcortical atlas (Desikan, Ségonne et al. 2006) and not by manual delineation. Note that the tracing of these regions only affected the visualization and labelling in the resulting connectograms and that the network mask used in the calculation was applied to the links and not the voxels.

**Figure 4:**
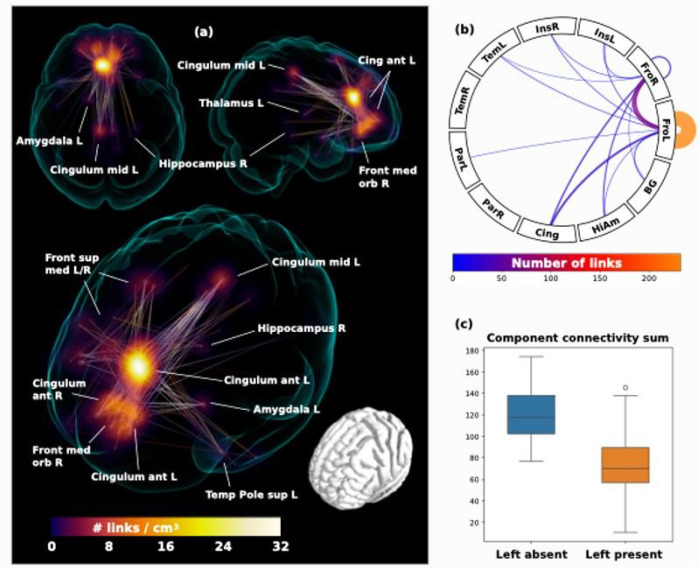
Voxel-based Connectivity Results Network component with significantly greater functional connectivity in the left Paracingulate Sulcus absent [Left absent] relative the Left Paracingulate Sulcus present [Left present] group (*P* = 0.01 controlled for family-wise error rate). The greatest link density (shown in voxel-wise maximal intensity projection in panel (a)) was found converging on the left anterior cingulum, extending inferiorly toward frontal medial orbitus and the right anterior cingulum. More extended connections were also found to frontal superior medial gyrus L/R and posterior cingulum, as well as scattered connections to subcortical structures: left amygdala, right posterior hippocampus, and left thalamus. Panel (b) shows the connectogram in the form of the number of links connecting the paracingulate high-connectivity regions (see Figure 3). (c) show a boxplot of the summed connectivities on the significantly differing network component for the [left absent] and [left present] groups.

In the second step, the whole brain functional connectome was limited to the identified regions connecting strongly to the ROIs and entered into a network based statistic component calculation (Zalesky, Fornito et al. 2010) comprising a connected set of links on which connectivity differed in accordance with ipsilateral hemispheric PCS presence, based on a binarized connectivity graph at a threshold of *P* < 0.001 (given group sizes), controlled for the effects of age, sex and handedness. Results of significant network components with altered connectivity and summarizing connectograms are displayed in Figure 4.

### Data Availability

Anonymized data will be shared by request from a qualified academic investigator for the sole purpose of replicating procedures and results presented in the article if data transfer is in agreement with relevant legislation on the general data protection regulation and decisions and by the relevant Ethical Review Boards, which should be regulated in a material transfer agreement.

## Results

### Tract Segmentation

**Figure 5:**
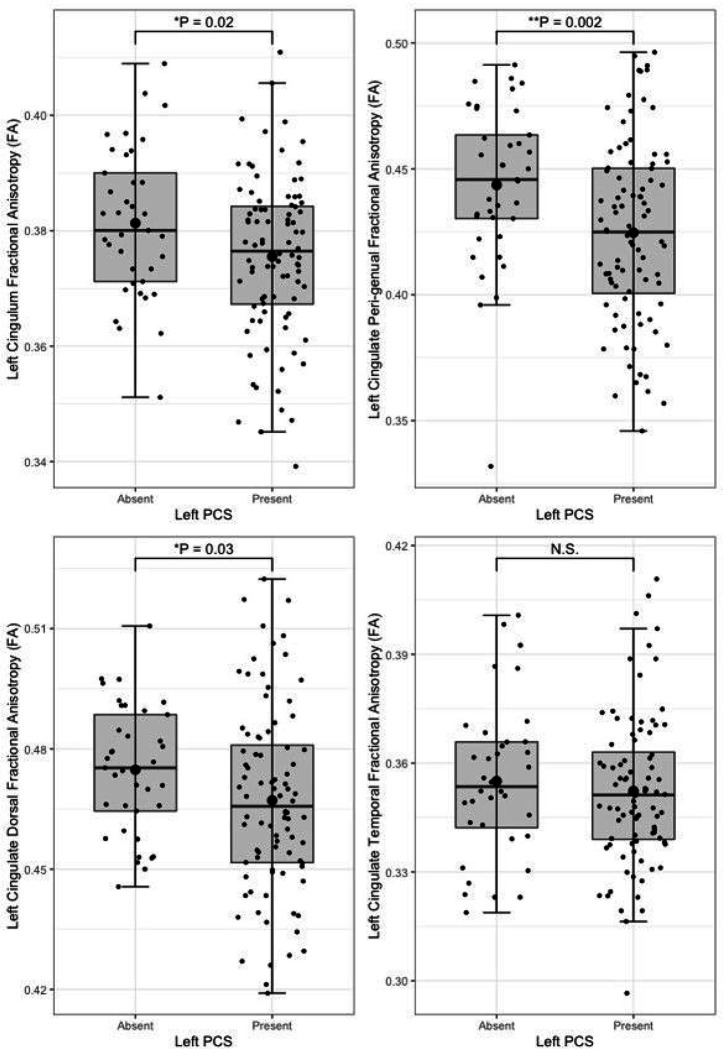
Cingulum bundle fractional anisotropy by left paracingulate sulcal presence Box plots displaying tract fractional anisotropy by left Paracingulate sulcal presence in the cingulum [TractSeg] (top left), peri-genual cingulum [Xtract] (top right), dorsal cingulum [Xtract] (bottom left) and temporal cingulum [Xtract] (bottom right). Black dots represent individuals, n = 125. Thick horizontal black lines represent group median values. Larger black dots represent group mean values. Boxes extend from the 25^th^ to the 75^th^ percentile, horizontal black lines within the boxes denote median values. P-values (P) of general linear models corrected for age, sex and handedness are displayed over the box plots. N.S. denotes no significant difference between groups. * Significance at P= < 0.05. ** P= < 0.01

**Figure 6:**
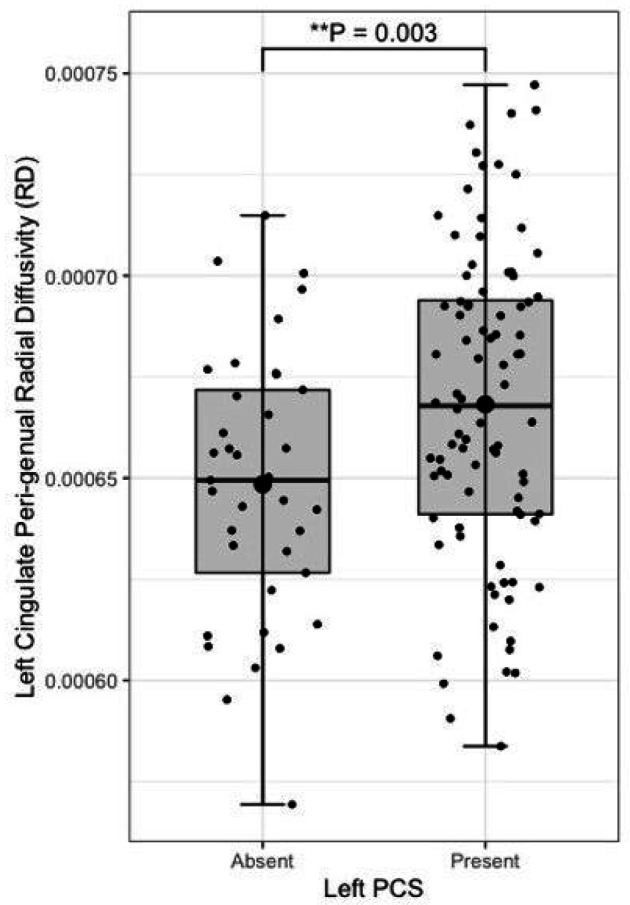
Peri-genual cingulum radial diffusivity by left paracingulate sulcal presence Box plot displaying left peri-genual cingulum [TractSeg] radial diffusivity by left Paracingulate sulcal presence. Black dots represent individuals, n = 125. Thick horizontal black lines represent group median values. Larger black dots represent group mean values. Boxes extend from the 25^th^ to the 75^th^ percentile, horizontal black lines within the boxes denote median values. P-values (P) of general linear models corrected for age, sex and handedness are displayed over the box plots. * Significance at P= < 0.05. ** P= < 0.01

Following quality control procedures segmentations were available for 125 subjects, (mean age 52.19, SD 5.12), see Table 1. The frequency of present to absent PCS was greater in the left (88/125) than right (71/125) hemisphere as expected. Individuals with a present left PCS displayed reduced FA of the left CG relative to individuals with an absent left PCS (β = −0.02, CI −0.01 - −0.0008 μm^2^/ms, *P* = 0.02). Using the Xtract method a present left PCS was associated with decreased FA in the ipsilateral CBG (β = −0.009, CI −0.04 - −0.008 μm^2^/ms, *P* = 0.002) and CBD (β = − 0.009, CI − 0.02 - − 0.0009 μm^2^/ms, *P* = 0.03) but not the CBT. These results are displayed in Fig. 5. Ipsilateral RD of the CBG was higher in individuals with a present left PCS compared to those with an absent left PCS (β = 2.22 ×10^− 5^, CI 7.58e-06-3.69e-05 μm^2^/ms, *P* = 0.003), see Fig. 6. Ipsilateral RD was similar according to PCS presence in the other studied tracts in both hemispheres.

**Figure 7:**
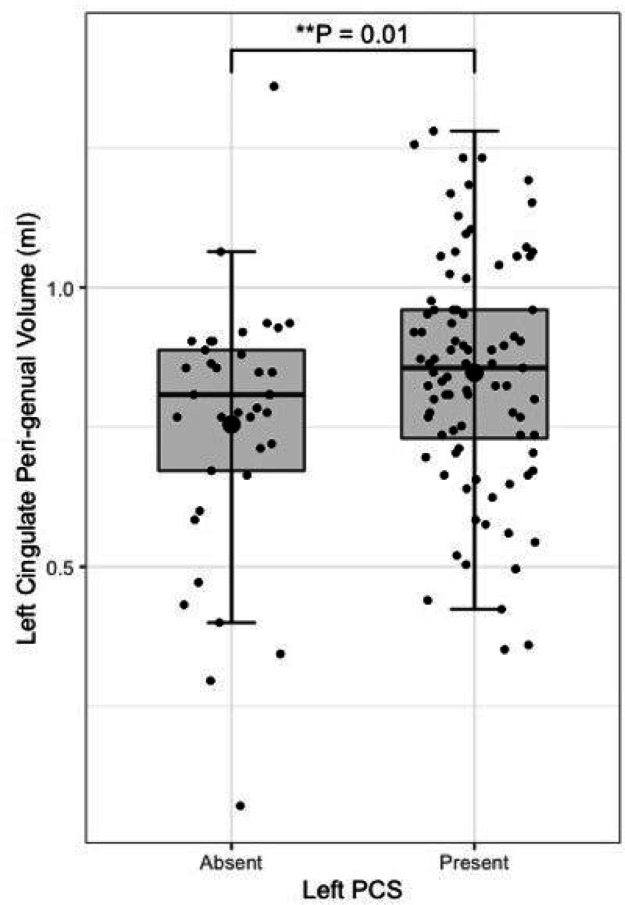
Peri-genual cingulum bundle tract volume by left paracingulate sulcal presence Box plot displaying left peri-genual cingulum [TractSeg] tract volume by left Paracingulate sulcal presence. Black dots represent individuals, n = 125. Thick horizontal black lines represent group median values. Larger black dots represent group mean values. Boxes extend from the 25^th^ to the 75^th^ percentile, horizontal black lines within the boxes denote median values. P-values (P) of general linear models corrected for age, sex and handedness are displayed over the box plots. * Significance at P= < 0.05. ** P= < 0.01

Left PCS presence was associated with increased left CBG tract volume (β = 0.10, CI 0.02–0.18 μm^2^/ms, *P* = 0.012), see Fig. 7. Right PCS presence was not associated with right CBG tract volume. SLF-I, CG, CBD, or CBT tract volume were not associated with ipsilateral left or right PCS presence. Results are displayed in full in Table 3.

### Functional Connectivity

Resting state fMRI data was available for 129 individuals, (mean age 52.46, SD 4.96), see Table 2.

### Seed-based Functional connectivity

Group wise intra-network resting state functional connectivity (rsFC) in ipsilateral hemispheric and whole brain analyses of all predefined networks was similar when comparing individuals with a present and absent left PCS and also individuals with a present and absent right PCS.

### Voxel-based Functional connectivity

Two individuals were excluded from analysis as they did not have available handedness data. Voxel based functional connectivity analyses were performed for 127 individuals. The only significant component was identified in individuals possessing a left PCS relative to individuals with an absent left PCS at *P* = 0.01, controlling for family wise error rate. Results are displayed in Fig. 4. The greatest link density was found converging on the left anterior cingulum, extending inferiorly towards the frontal medial orbitus and the right anterior cingulum. More extended connections were also found to the frontal superior medial gyrus, the left and right posterior cingulum, as well as scattered connections to subcortical structures including the left amygdala, the right posterior hippocampus, and left thalamus.

## Discussion

### Tract Segmentation

Results from the tract segmentation analyses indicate that absence of a left hemisphere PCS is associated with higher ipsilateral cingulate bundle FA. More specifically group diffusivity differences localise to the anterior portion of the cingulum; the peri-genual and dorsal cingulum bundles. Expectedly, no significant group diffusivity difference was observed in the offsite temporal division of the cingulate bundle. Furthermore, higher radial diffusivity and tract volume were observed in the left peri-genual cingulum bundle in individuals with a left PCS relative to those without. Ipsilateral tract volumes and diffusivity matrices were similar in the SLF-I between groups in both hemispheres. These results suggest that where a left PCS is present the ipsilateral cingulum bundle, specifically its anterior portions (peri-genual > dorsal) may display increased orientational dispersion. To the best of our knowledge these findings are novel and an association between gyrification and structural connectivity in healthy individuals has not previously been identified in the literature.

In the context of gyrification theories (Van Essen 1997, Van Essen 2020) we suggest that U-fibres, (short association fibres connecting adjacent gyri displaying a complex orientation relative to major long-white mater tracts) present in greater densities in individuals with a left PCS relative to those without may have influenced tract segment metrics. This suggestion is grounded by three principles: (1.) Inclusion of U-fibres in large tracts, referred to as a transverse inaccuracy contributes to increase the tract volume within a larger white matter tract and effect diffusivity (Jbabdi and Johansen-Berg 2011). (2.) U-fibres have lower orientational coherence resulting in lower FA values. Where U-fibres are incorporated into a major tract the overall orientational coherence therefore becomes lessened resulting in a lower FA. (3.) U-fibres follow the pattern of cortical folding and as such are orientated perpendicularly to the axonal fibres of the cingulum.(Movahedian Attar, Kirilina et al. 2020) U-fibre orientation and microstructure may therefore contribute to the observed increased RD in the CBP as water molecules diffuse more freely in a radial direction with respect to the CBP proper.

It is important to state that U-fibres are challenging to image and categorise due to their short length, size, and complex trajectories and though these metrics suggest their presence, a comprehensive assessment is indicated requiring ultra-high-resolution acquisitions as well as advanced imaging tractography methods specifically designed to identify and map U-fibres, which were not available in the present study.

In the context of the current literature our findings suggest that fibres impacted topographically by the presence of a PCS are more likely those of the cingulate bundle as suggested by Wu et al 2016 (Wu, Sun et al. 2016) than a segment of the SLF as suggested by Komaitis et al 2019 (Komaitis, Skandalakis et al. 2019). Tract segmentation analyses are however limited in comparison to dissection techniques by spatial resolution.

### Seed-based functional connectivity

Contrary to our hypothesis right PCS presence was not associated with rsFC strength in the predefined SN or DMN as assessed according to our seed-based rsfMRI approach. In turn the Visual network which acted as an off-site control in this study identified no group difference in rsFC suggesting that findings were not secondary to a type II error.

We believe these negative findings reflect the theory driven methodology in which network strength was analysed in accordance with parcellations corresponding to predefined networks mapped to MNI space. This method was coarse and reliant on consistent network topography between source networks used to derive the predefined networks and networks of individuals in our cohort regardless of PCS presence. Future seed-based study in this field may consider analysing network connectivity in networks derived directly form a voxel-based analysis of the same cohort.

### Voxel-based functional connectivity

We identify that the medial frontal lobe ROI corresponding with the location of the PCS is highly connected to regions of the cingulum, insula, frontal, temporal, and parietal cortex as well as the thalamus, caudate, pallidum, putamen, hippocampus, and amygdala, key components of the DMN and SN, in accordance with standard literature (Catani and Thiebaut de Schotten 2012). In the left hemisphere large-scale connectivity changes were observed revealing a significant network component with greater rsFC in individuals with an absent left PCS relative to those with a present left PCS. This component comprised the left and right anterior cingulum and frontal medial orbitus with more extended connections to the left and right frontal superior medial gyrus and the posterior cingulum, as well as scattered connections to subcortical structures; the left amygdala, the right posterior hippocampus, and left thalamus. The increase in connectivity identifies alternate functional architecture in individuals with an absent left PCS, where distributed network nodes are enlisted creating an alternate specialisation profile with auxiliary processing power drawn from more distal regions outside of the anterior cingulate. Again, with consideration of the findings from the tract segmentation analyses a more dispersed network as identified in individuals with an absent PCS, may become operational where a highly localised network (presumed to exist where a PCS is present) is not present. In turn, cognitive advantages reported in the literature (Fornito, Yücel et al. 2004, Whittle, Allen et al. 2009, Borst, Cachia et al. 2014, Cachia, Del Maschio et al. 2017) associated with the presence of a left PCS may be underpinned by an efficient highly localised network dependent on U-fibres rather than a well organised cingulum bundle. These observations are in line with and provide further evidence for the tension-based morphogenesis theory of cortical folding (Van Essen 1997) and support the notion that well interconnected brain regions display strong patterns of functional connectivity (Segall, Allen et al. 2012) (van den Heuvel, Stam et al. 2009). Extending this concept to disease, we speculate that a highly connected localised network existing in the presence of a PCS may account for the resilience to disease expression observed in individuals with a right PCS in bvFTD and explain why absence of a left PCS, a neurodevelopmental aberration, has been associated with schizophrenia and OCD (Harper, Lindberg et al. 2022) (Amiez, Neveu et al. 2013, Harper, de Boer et al. 2023) (Yücel, Stuart et al. 2002) (Shim, Jung et al. 2009). Studies exploring these hypotheses in these disease groups are indicated in order to provide evidence for this theory. Furthermore, it is known that AC gyral variability affects gyral volume and thus should be taken into account in the study of relevant diseases (Fornito, Whittle et al. 2006). Here we demonstrate that this is also the case for structural anatomy.

Contrary to findings of the present study, Fedeli et al 2020 explored rsFC with respect to PCS presence using a seed-based approach and did not identify an association between rsFC and left PCS presence (Fedeli, Del Maschio et al. 2020). Similarly to this study however, an association between individuals with absent PCS and enhanced long-distance rsFC was identified in Fedeli et al 2020. Albeit, this connection was formed with the cerebellum, a region not identified as a highly connected region to the medial frontal lobe ROI in our study and therefore not investigated further for connectivity differences according to PCS presence in the second part of our voxel-based analysis. Fedeli et al 2020 also report additional associations between whole brain PCS patterns (bilateral PCS status) and distinct profiles of rsFC. These findings include decreased connectivity in the insula in those with bilaterally absent PCS compared to those with bilaterally present PCS, extensive decreased patterns of long-distance rsFC to the bilateral occipital cortices, right temporo-occipital and cerebellar regions in individuals with a bilaterally absent PCS compared to those with a rightward dominant pattern and increased connectivity with the angular gyrus, insular and central opercular cortex in individuals with bilaterally present PCS compared to those with leftward dominant patterns (Fedeli, Del Maschio et al. 2020). Findings from this study indicate a functional effect of this gyral variation but lack a proposed unifying mechanistic theory. RsFC analysis in relation to whole brain PCS pattern was not performed in the present study due to powering though further study in this field should investigate this topic in order to identify if findings from Fedeli et al 2020 may be replicated. RsfMRI data in the present study was obtained at a spatial resolution of 6×6×6 mm^3^ voxel connectivity with 5000 voxels. Though collection of data at this resolution allows for timely attainment of data from large cohorts a potential limitation is that this degree of spatial resolution does not provide the sensitivity required to identify functional connectivity differences generated by highly localised networks. Furthermore, it is quite probable that both individuals with a present and absent PCS have inherently high intra-connectivity in a highly localized functional map within the targeted ROI which is not reflected in groupwise testing. Where feasible alternative fMRI techniques may be utilised for further exploration of this theory in future study.

### Laterality

We did not observe significant structural or functional connectivity differences in the right hemisphere consistent with those found in the left hemisphere in relation to PCS presence in this study. In terms of lateralisation there is a well-established asymmetry of the cingulum in studies based on diffusion MRI, such that volume (Takao, Hayashi et al. 2013) and FA, along the length the anterior cingulum displays a marked left-greater-than-right asymmetry (Gong, Jiang et al. 2005) (Park, Westin et al. 2004). Furthermore, superficial white matter, U-fibres, which contribute 90% of the total white matter fibres to the human brain, are known to display an asymmetrical distribution, with diffusivity matrices indicative of increased left hemisphere U-fibre structural integrity in the frontal, temporal, and parietal regions of healthy individuals (Movahedian Attar, Kirilina et al. 2020). With consideration of these data, the lateralisation identified in this study is most likely represented by the increased left-to-right hemisphere asymmetry in U-fibre density.

Lateralisation of the predefined functional networks was not identified here, however the SN is known to be organizationally dominant in the right hemisphere (Seeley, Menon et al. 2007, Zhang, Suo et al. 2019) with multimodal structural and functional imaging studies(Cauda, D’Agata et al. 2011, Zhang, Suo et al. 2019) (Seeley, Menon et al. 2007) (Zhang, Suo et al. 2019) identifying stronger and broader intrinsic functional network couplings in the right compared to left dorsal ACC. In turn, the right hemisphere SN has been identified to exhibit much weaker disassortativity (the degree of connection between nodes with low numbers of connections and nodes with high numbers of connections) than that of the left hemisphere (Lim, Radicchi et al. 2019). Similarly, these observations may also be explained by U-fibres density asymmetries between hemispheres.

### Summary

These results identify a novel association between sulcation, a neurodevelopmentally derived gross anatomical feature and altered structural and functional connectivity in a healthy adult population. Furthermore, they provide evidence of a link between structural and functional connectivity and a plausible explanation of how cognitive advantages of a paracingulate sulcus may be mediated by a highly connected local functional network reliant on short association fibres. The findings also have importance for understanding the neuropsychological aspects of this anatomical variation, and for understanding pathophysiology of the diseases in which this variation has a role. Additional work in this field utilizing multimodal imaging techniques in adequately sized cohorts is indicated to confirm results presented here, provide evidence to support our rational and investigate structural and functional connectivity with respect to whole brain PCS pattern.

## Figures and Tables

**Figure 8:**
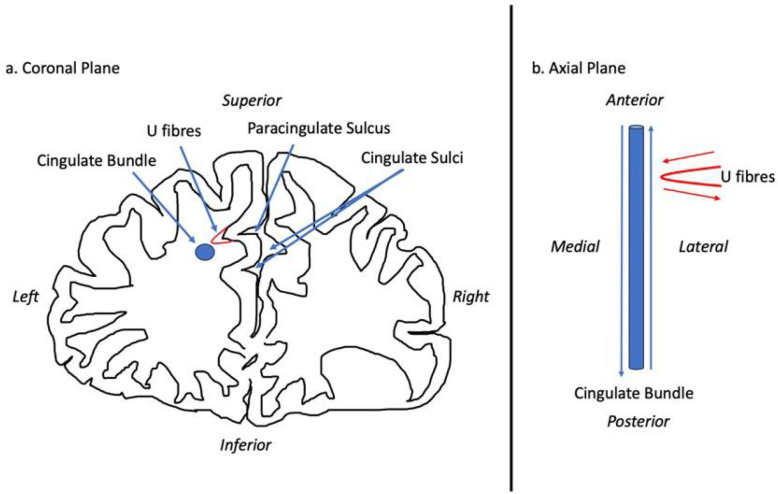
Schematic of the influence of a paracingulate sulcus on structural connectivity Panel (a) Schematic of a coronal cross-section of the frontal lobe of an individual with a present left and absent right Paracingulate sulcus. The blue circle represents a cross-section of the cingulum bundle along its axis. Adjacent perpendicular U-fibres are represented in red and are thought to be partially responsible for gyrification and the formation of a paracingulate sulcus. In panel (b) the schematic is appreciated from an axial plane. The cingulate bundle is represented in blue with blue arrows representing the predominant axial transmission along the length of the bundle. U fibres are represented in red with transmission along fibres represented by red arrows. Transmission along the cingulate bundle and adjacent U-fibres occur perpendicular to one another. Where there are increased U-fibres, such as in the presence of a paracingulate sulcus we may assume that U-fibres adjacent to the cingulate bundle may be attributed to the bundle increasing its volume and radial diffusivity increases whilst decreasing it’s fractional anisotropy.

**Table 1:** Structural Connectivity Study Population & Paracingulate Sulcal Status

	Total	Left PCS Present	Left PCS Absent	Left PCS Contrasts	Right PCS Present	Right PCS Absent	Right PCS Contrasts
n	125	88	37		71	54	
Age, mean (SD), years	52.19 (5.12)	52.37 (5.06)	51.76 (5.33)	F = 0.36, *P* = 0.55	51.63 (4.95)	52.92 (5.30)	F = 1.94, *P* = 0.17
Sex				X^2^ = 3.15, *P* = 0.08			X^2^ = 1.95, *P* = 0.16
Female	71	45	26		36	36	
Male	54	43	11		35	19	
Handedness^a^				X^2^ = 1.09, *P* = 0.58			X^2^ = 1.67, *P* = 0.43
Right	113	80	33		64	49	
Left	8	5	3		4	4	
Ambidextrous	2	2	0		2	0	
Unknown	2	1	1		1	1	

Demographic data for the structural connectivity analyses.

Standard deviation (SD).

a Handedness data available for 123/125 individuals. Hemispheric Paracingulate Sulcal Status; present = PCS length ≥ 20mm. ANOVA and Chi-Squared tests were performed to evaluate differences in continuous and nominal data, respectively.

**Table 2:** Functional Connectivity Study Population & Paracingulate Sulcal Status

	Total	Left PCS Present	Left PCS Absent	Left PCS Contrasts	Right PCS Present	Right PCS Absent	Right PCS Contrasts
n	129	92	37		74	55	
Age, mean (SD), years	52.46 (4.96)	52.59 (4.95)	52.14 (5.05)	F = 0.22, *P* = 0.64	51.82 (4.79)	53.32 (5.11)	F = 2.95, *P* = 0.08
Sex				X^2^ = 3.61, *P* = 0.06			X^2^ = 1.00, *P* = 0.32
Female	72	46	26		38	34	
Male	57	46	11		36	21	
Handedness^a^				X^2^ = 0.83, *P* = 0.66			X^2^ = 2.11, *P* = 0.35
Right	116	83	33		67	49	
Left	9	6	3		4	5	
Ambidextrous	2	2	3		2	0	
Unknown	2	1	1		1	1	

Demographic data for the functional connectivity analyses.

Standard deviation (SD).

a Handedness data available for 127/129 individuals. Hemispheric Paracingulate Sulcal Status; present = PCS length ≥ 20mm. ANOVA and Chi-Squared tests were performed to evaluate differences in continuous and nominal data, respectively.

**Table 3:** Structural Connectivity Results

Tract	Segmentation method	Tract Volume	Fractional Anisotropy	Mean Diffusivity	Radial Diffusivity	Axial Diffusivity
Cingulum bundle, left	TractSeg	β = 0.50, *P* = 0.53	β = −6.3e-03, *P* = 0.02*	β = 6.0e-06, *P* = 0.17	β = 8.0e-06, *P* = 0.09	β = 1.9e-06, *P* = 0.65
Cingulum bundle, right	TractSeg	β = 0.59, *P* = 0.30	β = −4.2e-04, *P* = 0.87	β = −4.7e-07, *P* = 0.91	β = −5.7e-07, *P* = 0.90	β = −2.6e-07, *P* = 0.95
Peri-genual cingulum, left	Xtract	β = 0.09, *P* = 0.01*	β = −0.02, *P* = 0.002*	β = 1.1e-05, *P* = 0.10	β = 2.2e-05, *P* = 0.003*	β = −1.1e-05, *P* = 0.39
Peri-genual cingulum, right	Xtract	β = −0.05, *P* = 0.28	β = 2.2e-03, *P* = 0.72	β = −6.4e-06, *P* = 0.33	β = −6.4e-06, *P* = 0.33	β = −3.4e-07, *P* = 0.97
Cingulum bundle dorsal, left	Xtract	β = 0.29, *P* = 0.07	β = −9.3 e-03, *P* = 0.03*	β = 5.7e-06, *P* = 0.24	β = 6.7e-06, *P* = 0.09	β = −2.2e-06, *P* = 0.72
Cingulum bundle dorsal, right	Xtract	β = 0.03, *P* = 0.82	β = −4.5 e-03, *P* = 0.28	β = −3.6e-07, *P* = 0.94	β = 1.9e-06, *P* = 0.73	β = −4.9e-06, *P* = 0.43
Cingulum bundle temporal, left	Xtract	β = 0.11, *P* = 0.33	β = −2.9 e-03, *P* = 0.48	β = 1.6e-05, *P* = 0.04*	β = 1.6e-05, *P* = 0.04*	β = 1.6e-05, *P* = 0.10
Cingulum bundle temporal, right	Xtract	β = 0.02, *P* = 0.83	β = −3.5 e-03, *P* = 0.47	β = 3.0e-07, *P* = 0.97	β = 2.2e-06, *P* = 0.77	β = 1.6e-05, *P* = 0.10
Superior longitudinal fasciculus I, left	TractSeg	β = −0.81,*P* = 0.21	β = 5.5e-06, *P* = 0.25	β = 7.1e-06, *P* = 0.18	β = 7.1e-06, *P* = 0.18	β = 2.3e-06, *P* = 0.67
Superior longitudinal fasciculus I, right	TractSeg	β = 0.29, *P* = 0.71	β = 1.6e-04, *P* = 0.96	β = −3.7e-06, *P* = 0.41	β = −3.4e-06, *P* = 0.49	β = −3.5e-06, *P* = 0.71
Superior longitudinal fasciculus I, left	Xtract	β = 0.12, *P* = 0.61	β = −5.9e-03, *P* = 0.23	β = 4.7e-06, *P* = 0.44	β = 6.6e-06, *P* = 0.35	β = 8.6e-06, *P* = 0.90
Superior longitudinal fasciculus I, right	Xtract	β = 0.22, *P* = 0.34	β = −2.1e-03, *P* = 0.63	β = −4.8e-06, *P* = 0.39	β = −2.2e-06, *P* = 0.73	β = −1.0e-05, *P* = 0.18

General Linear Models for differences in tract structural connectivity matrices according to ipsilateral paracingulate sulcal presence. All models are corrected for age, sex, and handedness. Model of tract volume are additionally corrected for total intracranial volume.

* Denotes significance at *P* < 0.05.

## References

[1] American Psychiatric Association (2013). Diagnostic and Statistical Manual of Mental Disorders. Washington, DC.

[2] Amiez C., Neveu R., Warrot D., Petrides M., Knoblauch K. and Procyk E. (2013). “The Location of Feedback-Related Activity in the Midcingulate Cortex Is Predicted by Local Morphology.” The Journal of Neuroscience 33(5): 2217–2228. 23365257 10.1523/JNEUROSCI.2779-12.2013 PMC6619107

[3] Amiez C., Wilson C. R. and Procyk E. (2018). “Variations of cingulate sulcal organization and link with cognitive performance.” Scientific Reports 8(1): 1–13. 30228357 10.1038/s41598-018-32088-9 PMC6143647

[4] Andersson J. L., Skare S. and Ashburner J. (2003). “How to correct susceptibility distortions in spin-echo echo-planar images: application to diffusion tensor imaging.” Neuroimage 20(2): 870–888. 14568458 10.1016/S1053-8119(03)00336-7

[5] Andersson J. L. R. and Sotiropoulos S. N. (2016). “An integrated approach to correction for off-resonance effects and subject movement in diffusion MR imaging.” Neuroimage 125: 1063–1078. 26481672 10.1016/j.neuroimage.2015.10.019 PMC4692656

[6] Avants B. B., Tustison N. J., Stauffer M., Song G., Wu B. and Gee J. C. (2014). “The Insight ToolKit image registration framework.” Frontiers in neuroinformatics 8: 44. 24817849 10.3389/fninf.2014.00044 PMC4009425

[7] Behzadi Y., Restom K., Liau J. and Liu T. T. (2007). “A component based noise correction method (CompCor) for BOLD and perfusion based fMRI.” Neuroimage 37(1): 90–101. 17560126 10.1016/j.neuroimage.2007.04.042 PMC2214855

[8] Borst G., Cachia A., Vidal J., Simon G., Fischer C., Pineau A., Poirel N., Mangin J. F. and Houdé O. (2014). “Folding of the anterior cingulate cortex partially explains inhibitory control during childhood: a longitudinal study.” Dev Cogn Neurosci 9: 126–135. 24642370 10.1016/j.dcn.2014.02.006 PMC6989755

[9] Borst G., Cachia A., Vidal J., Simon G., Fischer C., Pineau A., Poirel N., Mangin J. F. and Houdé O. (2014). “Folding of the anterior cingulate cortex partially explains inhibitory control during childhood: A longitudinal study.” Developmental Cognitive Neuroscience 9: 126–135. 24642370 10.1016/j.dcn.2014.02.006 PMC6989755

[10] Buda M., Fornito A., Bergstrom Z. M. and Simons J. S. (2011). “A specific brain structural basis for individual differences in reality monitoring.” J Neurosci 31(40): 14308–14313. 21976516 10.1523/JNEUROSCI.3595-11.2011 PMC3190297

[11] Cachia A., Borst G., Vidal J., Fischer C., Pineau A., Mangin J.-F. and Houdé O. (2014). “The Shape of the ACC Contributes to Cognitive Control Efficiency in Preschoolers.” Journal of Cognitive Neuroscience 26(1): 96–106. 23915057 10.1162/jocn_a_00459

[12] Cachia A., Del Maschio N., Borst G., Della Rosa P. A., Pallier C., Costa A., Houdé O. and Abutalebi J. (2017). “Anterior cingulate cortex sulcation and its differential effects on conflict monitoring in bilinguals and monolinguals.” Brain Lang 175: 57–63. 29017088 10.1016/j.bandl.2017.09.005

[13] Catani M. and Thiebaut de Schotten M. (2012). Atlas of Human Brain Connections, Oxford University Press.

[14] Cauda F., D’Agata F., Sacco K., Duca S., Geminiani G. and Vercelli A. (2011). “Functional connectivity of the insula in the resting brain.” Neuroimage 55(1): 8–23. 21111053 10.1016/j.neuroimage.2010.11.049

[15] Chi J. G., Dooling E. C. and Gilles F. H. (1977). “Gyral development of the human brain.” Ann Neurol 1(1): 86–93. 560818 10.1002/ana.410010109

[16] Cox R. W. (1996). “AFNI: software for analysis and visualization of functional magnetic resonance neuroimages.” Computers and Biomedical research 29(3): 162–173. 8812068 10.1006/cbmr.1996.0014

[17] Del Maschio N., Sulpizio S., Fedeli D., Ramanujan K., Ding G., Weekes B. S., Cachia A. and Abutalebi J. (2019). “ACC Sulcal Patterns and Their Modulation on Cognitive Control Efficiency Across Lifespan: A Neuroanatomical Study on Bilinguals and Monolinguals.” Cereb Cortex 29(7): 3091–3101. 30059975 10.1093/cercor/bhy175

[18] Desikan R. S., Ségonne F., Fischl B., Quinn B. T., Dickerson B. C., Blacker D., Buckner R. L., Dale A. M., Maguire R. P. and Hyman B. T. (2006). “An automated labeling system for subdividing the human cerebral cortex on MRI scans into gyral based regions of interest.” Neuroimage 31(3): 968–980. 16530430 10.1016/j.neuroimage.2006.01.021

[19] Farb N. A., Grady C. L., Strother S., Tang-Wai D. F., Masellis M., Black S., Freedman M., Pollock B. G., Campbell K. L., Hasher L. and Chow T. W. (2013). “Abnormal network connectivity in frontotemporal dementia: evidence for prefrontal isolation.” Cortex 49(7): 1856–1873. 23092697 10.1016/j.cortex.2012.09.008

[20] Fedeli D., Del Maschio N., Caprioglio C., Sulpizio S. and Abutalebi J. (2020). “Sulcal Pattern Variability and Dorsal Anterior Cingulate Cortex Functional Connectivity Across Adult Age.” Brain Connect 10(6): 267–278. 32567343 10.1089/brain.2020.0751

[21] Fedeli D., Del Maschio N., Del Mauro G., Defendenti F., Sulpizio S. and Abutalebi J. (2022). “Cingulate cortex morphology impacts on neurofunctional activity and behavioral performance in interference tasks.” Scientific Reports 12(1): 13684. 35953536 10.1038/s41598-022-17557-6 PMC9372177

[22] Fornito A., Whittle S., Wood S. J., Velakoulis D., Pantelis C. and Yücel M. (2006). “The influence of sulcal variability on morphometry of the human anterior cingulate and paracingulate cortex.” Neuroimage 33(3): 843–854. 16996751 10.1016/j.neuroimage.2006.06.061

[23] Fornito A., Yücel M., Wood S., Stuart G. W., Buchanan J.-A., Proffitt T., Anderson V., Velakoulis D. and Pantelis C. (2004). “Individual Differences in Anterior Cingulate/Paracingulate Morphology Are Related to Executive Functions in Healthy Males.” Cerebral Cortex 14(4): 424–431. 15028646 10.1093/cercor/bhh004

[24] Fujiwara H., Hirao K., Namiki C., Yamada M., Shimizu M., Fukuyama H., Hayashi T. and Murai T. (2007). “Anterior cingulate pathology and social cognition in schizophrenia: a study of gray matter, white matter and sulcal morphometry.” Neuroimage 36(4): 1236–1245. 17524666 10.1016/j.neuroimage.2007.03.068

[25] Garrison J. R., Fernyhough C., McCarthy-Jones S., Haggard M. and Simons J. S. (2015). “Paracingulate sulcus morphology is associated with hallucinations in the human brain.” Nat Commun 6: 8956. 26573408 10.1038/ncomms9956 PMC4660352

[26] Gong G., Jiang T., Zhu C., Zang Y., Wang F., Xie S., Xiao J. and Guo X. (2005). “Asymmetry analysis of cingulum based on scale-invariant parameterization by diffusion tensor imaging.” Hum Brain Mapp 24(2): 92–98. 15455461 10.1002/hbm.20072 PMC6871701

[27] Grabner G., Janke A. L., Budge M. M., Smith D., Pruessner J. and Collins D. L. (2006). Symmetric atlasing and model based segmentation: an application to the hippocampus in older adults. Medical Image Computing and Computer-Assisted Intervention–MICCAI 2006: 9th International Conference, Copenhagen, Denmark, October 1–6, 2006. Proceedings, Part II 9, Springer. 10.1007/11866763_8 17354756

[28] Harper L., de Boer S., Lindberg O., Lätt J., Cullen N., Clark L., Irwin D., Massimo L., Grossman M., Hansson O., Pijnenburg Y., McMillan C. T. and Santillo A. F. (2023). “Anterior cingulate sulcation is associated with onset and survival in frontotemporal dementia.” Brain Communications 5(5). 10.1093/braincomms/fcad264 PMC10586312 37869576

[29] Harper L., Lindberg O., Bocchetta M., Todd E. G., Strandberg O., van Westen D., Stomrud E., Landqvist Waldö M., Wahlund L.-O., Hansson O., Rohrer J. D. and Santillo A. (2022). “Prenatal gyrification pattern affects age at onset in frontotemporal dementia.” Cerebral Cortex 32(18): 3937–3944. 35034126 10.1093/cercor/bhab457 PMC9476616

[30] Harper L., Lindberg O., Bocchetta M., Todd E. G., Strandberg O., van Westen D., Stomrud E., Landqvist Waldö M., Wahlund L.-O., Hansson O., Rohrer J. D. and Santillo A. (2022). “Prenatal Gyrification Pattern Affects Age at Onset in Frontotemporal Dementia.” Cerebral Cortex. 10.1093/cercor/bhab457 PMC9476616 35034126

[31] Henriques R. N., Correia M. M., Marrale M., Huber E., Kruper J., Koudoro S., Yeatman J. D., Garyfallidis E. and Rokem A. (2021). “Diffusional Kurtosis Imaging in the Diffusion Imaging in Python Project.” Front Hum Neurosci 15: 675433. 34349631 10.3389/fnhum.2021.675433 PMC8327208

[32] Huster R. J., Westerhausen R., Kreuder F., Schweiger E. and Wittling W. (2007). “Morphologic asymmetry of the human anterior cingulate cortex.” Neuroimage 34(3): 888–895. 17161625 10.1016/j.neuroimage.2006.10.023

[33] Huster R. J., Wolters C., Wollbrink A., Schweiger E., Wittling W., Pantev C. and Junghofer M. (2009). “Effects of anterior cingulate fissurization on cognitive control during stroop interference.” Hum Brain Mapp 30(4): 1279–1289. 18570202 10.1002/hbm.20594 PMC6870757

[34] Jahn A., Nee D. E., Alexander W. H. and Brown J. W. (2016). “Distinct Regions within Medial Prefrontal Cortex Process Pain and Cognition.” J Neurosci 36(49): 12385–12392. 27807031 10.1523/JNEUROSCI.2180-16.2016 PMC5148227

[35] Jbabdi S. and Johansen-Berg H. (2011). “Tractography: where do we go from here?” Brain Connect 1(3): 169–183. 22433046 10.1089/brain.2011.0033 PMC3677805

[36] Jenkinson M., Beckmann C. F., Behrens T. E., Woolrich M. W. and Smith S. M. (2012). “Fsl.” Neuroimage 62(2): 782–790. 21979382 10.1016/j.neuroimage.2011.09.015

[37] Johnstone T., Ores Walsh K. S., Greischar L. L., Alexander A. L., Fox A. S., Davidson R. J. and Oakes T. R. (2006). “Motion correction and the use of motion covariates in multiple-subject fMRI analysis.” Human brain mapping 27(10): 779–788. 16456818 10.1002/hbm.20219 PMC6871380

[38] Kay B. P., Holland S. K., Privitera M. D. and Szaflarski J. P. (2014). “Differences in paracingulate connectivity associated with epileptiform discharges and uncontrolled seizures in genetic generalized epilepsy.” Epilepsia 55(2): 256–263. 24447031 10.1111/epi.12486 PMC4045634

[39] Komaitis S., Skandalakis G. P., Kalyvas A. V., Drosos E., Lani E., Emelifeonwu J., Liakos F., Piagkou M., Kalamatianos T., Stranjalis G. and Koutsarnakis C. (2019). “Dorsal component of the superior longitudinal fasciculus revisited: novel insights from a focused fiber dissection study.” J Neurosurg 132(4): 1265–1278. 30835690 10.3171/2018.11.JNS182908

[40] Le Provost J. B., Bartres-Faz D., Paillere-Martinot M. L., Artiges E., Pappata S., Recasens C., Perez-Gomez M., Bernardo M., Baeza I., Bayle F. and Martinot J. L. (2003). “Paracingulate sulcus morphology in men with early-onset schizophrenia.” Br J Psychiatry 182: 228–232. 12611786 10.1192/bjp.182.3.228

[41] Leonard C. M., Towler S., Welcome S. and Chiarello C. (2009). “Paracingulate asymmetry in anterior and midcingulate cortex: sex differences and the effect of measurement technique.” Brain Struct Funct 213(6): 553–569. 19636589 10.1007/s00429-009-0210-z

[42] Lim S., Radicchi F., van den Heuvel M. P. and Sporns O. (2019). “Discordant attributes of structural and functional brain connectivity in a two-layer multiplex network.” Sci Rep 9(1): 2885. 30814615 10.1038/s41598-019-39243-w PMC6393555

[43] Maldonado I. L., Mandonnet E. and Duffau H. (2012). “Dorsal fronto-parietal connections of the human brain: a fiber dissection study of their composition and anatomical relationships.” Anat Rec (Hoboken) 295(2): 187–195. 22190345 10.1002/ar.21533

[44] Marquardt R. K., Levitt J. G., Blanton R. E., Caplan R., Asarnow R., Siddarth P., Fadale D., McCracken J. T. and Toga A. W. (2005). “Abnormal development of the anterior cingulate in childhood-onset schizophrenia: a preliminary quantitative MRI study.” Psychiatry Research: Neuroimaging 138(3): 221–233. 10.1016/j.pscychresns.2005.01.001 15854790

[45] Movahedian Attar F., Kirilina E., Haenelt D., Pine K. J., Trampel R., Edwards L. J. and Weiskopf N. (2020). “Mapping Short Association Fibers in the Early Cortical Visual Processing Stream Using In Vivo Diffusion Tractography.” Cereb Cortex 30(8): 4496–4514. 32297628 10.1093/cercor/bhaa049 PMC7325803

[46] Ono M., Kubik S. and Abernathey C. D. (1990). “Atlas of the cerebral sulci.”

[47] Palmqvist S., Janelidze S., Quiroz Y. T., Zetterberg H., Lopera F., Stomrud E., Su Y., Chen Y., Serrano G. E., Leuzy A., Mattsson-Carlgren N., Strandberg O., Smith R., Villegas A., Sepulveda-Falla D., Chai X., Proctor N. K., Beach T. G., Blennow K., Dage J. L., Reiman E. M. and Hansson O. (2020). “Discriminative Accuracy of Plasma Phospho-tau217 for Alzheimer Disease vs Other Neurodegenerative Disorders.” Jama 324(8): 772–781. 32722745 10.1001/jama.2020.12134 PMC7388060

[48] Park H. J., Westin C. F., Kubicki M., Maier S. E., Niznikiewicz M., Baer A., Frumin M., Kikinis R., Jolesz F. A., McCarley R. W. and Shenton M. E. (2004). “White matter hemisphere asymmetries in healthy subjects and in schizophrenia: a diffusion tensor MRI study.” Neuroimage 23(1): 213–223. 15325368 10.1016/j.neuroimage.2004.04.036 PMC2794419

[49] Paus T., Tomaiuolo F., Otaky N., MacDonald D., Petrides M., Atlas J., Morris R. and Evans A. C. (1996). “Human cingulate and paracingulate sulci: pattern, variability, asymmetry, and probabilistic map.” Cereb Cortex 6(2): 207–214. 8670651 10.1093/cercor/6.2.207

[50] Power J. D., Barnes K. A., Snyder A. Z., Schlaggar B. L. and Petersen S. E. (2012). “Spurious but systematic correlations in functional connectivity MRI networks arise from subject motion.” Neuroimage 59(3): 2142–2154. 22019881 10.1016/j.neuroimage.2011.10.018 PMC3254728

[51] Schaefer A., Kong R., Gordon E. M., Laumann T. O., Zuo X.-N., Holmes A. J., Eickhoff S. B. and Yeo B. T. T. (2017). “Local-Global Parcellation of the Human Cerebral Cortex from Intrinsic Functional Connectivity MRI.” Cerebral Cortex 28(9): 3095–3114. 10.1093/cercor/bhx179 PMC6095216 28981612

[52] Schaefer A., Kong R., Gordon E. M., Laumann T. O., Zuo X. N., Holmes A. J., Eickhoff S. B. and Yeo B. T. T. (2018). “Local-Global Parcellation of the Human Cerebral Cortex from Intrinsic Functional Connectivity MRI.” Cereb Cortex 28(9): 3095–3114. 28981612 10.1093/cercor/bhx179 PMC6095216

[53] Seeley W. W., Menon V., Schatzberg A. F., Keller J., Glover G. H., Kenna H., Reiss A. L. and Greicius M. D. (2007). “Dissociable intrinsic connectivity networks for salience processing and executive control.” J Neurosci 27(9): 2349–2356. 17329432 10.1523/JNEUROSCI.5587-06.2007 PMC2680293

[54] Segall J. M., Allen E. A., Jung R. E., Erhardt E. B., Arja S. K., Kiehl K. and Calhoun V. D. (2012). “Correspondence between structure and function in the human brain at rest.” Front Neuroinform 6: 10. 22470337 10.3389/fninf.2012.00010 PMC3313067

[55] Selahi Ö., Kuru Bektaşoğlu P., Hakan T., Firat Z., Güngör A. and Çelikoğlu E. (2022). “Cingulate sulcus morphology and paracingulate sulcus variations: Anatomical and radiological studies.” Clin Anat. 10.1002/ca.23981 36403099

[56] Shim G., Jung W. H., Choi J. S., Jung M. H., Jang J. H., Park J. Y., Choi C. H., Kang D. H. and Kwon J. S. (2009). “Reduced cortical folding of the anterior cingulate cortex in obsessive-compulsive disorder.” J Psychiatry Neurosci 34(6): 443–449. 19949720 PMC2783435

[57] Szaflarski J. P., Kay B., Gotman J., Privitera M. D. and Holland S. K. (2013). “The relationship between the localization of the generalized spike and wave discharge generators and the response to valproate.” Epilepsia 54(3): 471–480. 23294001 10.1111/epi.12062 PMC3920282

[58] Takao H., Hayashi N. and Ohtomo K. (2013). “White matter microstructure asymmetry: Effects of volume asymmetry on fractional anisotropy asymmetry.” Neuroscience 231: 1–12. 23219841 10.1016/j.neuroscience.2012.11.038

[59] Thomas Yeo B., Krienen F. M., Sepulcre J., Sabuncu M. R., Lashkari D., Hollinshead M., Roffman J. L., Smoller J. W., Zöllei L. and Polimeni J. R. (2011). “The organization of the human cerebral cortex estimated by intrinsic functional connectivity.” Journal of neurophysiology 106(3): 1125–1165. 21653723 10.1152/jn.00338.2011 PMC3174820

[60] Tissier C., Linzarini A., Allaire-Duquette G., Mevel K., Poirel N., Dollfus S., Etard O., Orliac F., Peyrin C., Charron S., Raznahan A., Houdé O., Borst G. and Cachia A. (2018). “Sulcal Polymorphisms of the IFC and ACC Contribute to Inhibitory Control Variability in Children and Adults.” eNeuro 5(1). 10.1523/ENEURO.0197-17.2018 PMC5844057 29527565

[61] Tournier J. D., Smith R., Raffelt D., Tabbara R., Dhollander T., Pietsch M., Christiaens D., Jeurissen B., Yeh C. H. and Connelly A. (2019). “MRtrix3: A fast, flexible and open software framework for medical image processing and visualisation.” Neuroimage 202: 116137. 31473352 10.1016/j.neuroimage.2019.116137

[62] van den Heuvel M. P., Stam C. J., Kahn R. S. and Hulshoff Pol H. E. (2009). “Efficiency of functional brain networks and intellectual performance.” J Neurosci 29(23): 7619–7624. 19515930 10.1523/JNEUROSCI.1443-09.2009 PMC6665421

[63] Van Essen D. C. (1997). “A tension-based theory of morphogenesis and compact wiring in the central nervous system.” Nature 385(6614): 313–318. 9002514 10.1038/385313a0

[64] Van Essen D. C. (2020). “A 2020 view of tension-based cortical morphogenesis.” Proc Natl Acad Sci U S A. 10.1073/pnas.2016830117 PMC7780001 33323481

[65] Vogt B. A., Nimchinsky E. A., Vogt L. J. and Hof P. R. (1995). “Human cingulate cortex: surface features, flat maps, and cytoarchitecture.” J Comp Neurol 359(3): 490–506. 7499543 10.1002/cne.903590310

[66] Warrington S., Bryant K. L., Khrapitchev A. A., Sallet J., Charquero-Ballester M., Douaud G., Jbabdi S., Mars R. B. and Sotiropoulos S. N. (2020). “XTRACT - Standardised protocols for automated tractography in the human and macaque brain.” Neuroimage 217: 116923. 32407993 10.1016/j.neuroimage.2020.116923 PMC7260058

[67] Wasserthal J., Neher P. and Maier-Hein K. H. (2018). “TractSeg - Fast and accurate white matter tract segmentation.” Neuroimage 183: 239–253. 30086412 10.1016/j.neuroimage.2018.07.070

[68] Wei X., Yin Y., Rong M., Zhang J., Wang L., Wu Y., Cai Q., Yu C., Wang J. and Jiang T. (2017) “Paracingulate Sulcus Asymmetry in the Human Brain: Effects of Sex, Handedness, and Race.” Scientific reports 7, 42033 DOI: 10.1038/srep42033. 28195205 PMC5307317

[69] Whittle S., Allen N. B., Fornito A., Lubman D. I., Simmons J. G., Pantelis C. and Yücel M. (2009). “Variations in cortical folding patterns are related to individual differences in temperament.” Psychiatry Res 172(1): 68–74. 19250804 10.1016/j.pscychresns.2008.06.005

[70] Whittle S., Allen N. B., Fornito A., Lubman D. I., Simmons J. G., Pantelis C. and Yücel M. (2009). “Variations in cortical folding patterns are related to individual differences in temperament.” Psychiatry Research: Neuroimaging 172(1): 68–74. 10.1016/j.pscychresns.2008.06.005 19250804

[71] Wu Y., Sun D., Wang Y., Wang Y. and Ou S. (2016). “Segmentation of the Cingulum Bundle in the Human Brain: A New Perspective Based on DSI Tractography and Fiber Dissection Study.” Frontiers in Neuroanatomy 10. 10.3389/fnana.2016.00084 PMC5013069 27656132

[72] Wysiadecki G., Mazurek A., Walocha J., Majos A., Tubbs R. S., Iwanaga J., Żytkowski A. and Radek M. (2021). “Revisiting the Morphology and Classification of the Paracingulate Gyrus with Commentaries on Ambiguous Cases.” Brain Sci 11(7). 10.3390/brainsci11070872 PMC8301833 34210078

[73] Yagmurlu K., Middlebrooks E. H., Tanriover N. and Rhoton A. L. Jr. (2016). “Fiber tracts of the dorsal language stream in the human brain.” J Neurosurg 124(5): 1396–1405. 26587654 10.3171/2015.5.JNS15455

[74] Yücel M., Stuart G. W., Maruff P., Velakoulis D., Crowe S. F., Savage G. and Pantelis C. (2001). “Hemispheric and gender-related differences in the gross morphology of the anterior cingulate/paracingulate cortex in normal volunteers: an MRI morphometric study.” Cereb Cortex 11(1): 17–25. 11113032 10.1093/cercor/11.1.17

[75] Yücel M., Stuart G. W., Maruff P., Wood S. J., Savage G. R., Smith D. J., Crowe S. F., Copolov D. L., Velakoulis D. and Pantelis C. (2002). “Paracingulate morphologic differences in males with established schizophrenia: a magnetic resonance imaging morphometric study.” Biol Psychiatry 52(1): 15–23. 12079726 10.1016/s0006-3223(02)01312-4

[76] Yücel M., Wood S. J., Phillips L. J., Stuart G. W., Smith D. J., Yung A., Velakoulis D., McGorry P. D. and Pantelis C. (2003). “Morphology of the anterior cingulate cortex in young men at ultra-high risk of developing a psychotic illness.” The British Journal of Psychiatry 182(6): 518–524. 12777343 10.1192/bjp.182.6.518

[77] Zalesky A., Fornito A. and Bullmore E. T. (2010). “Network-based statistic: identifying differences in brain networks.” Neuroimage 53(4): 1197–1207. 20600983 10.1016/j.neuroimage.2010.06.041

[78] Zhang Y., Suo X., Ding H., Liang M., Yu C. and Qin W. (2019). “Structural connectivity profile supports laterality of the salience network.” Hum Brain Mapp 40(18): 5242–5255. 31436006 10.1002/hbm.24769 PMC6864895

